# Prediction of liver cancer prognosis based on immune cell marker genes

**DOI:** 10.3389/fimmu.2023.1147797

**Published:** 2023-04-27

**Authors:** Jianfei Liu, Junjie Qu, Lingling Xu, Chen Qiao, Guiwen Shao, Xin Liu, Hui He, Jian Zhang

**Affiliations:** ^1^ Department of Interventional Therapy, The First Affiliated Hospital of Dalian Medical University, Dalian, Liaoning, China; ^2^ Interventional Medicine Center, Affiliated Zhongshan Hospital of Dalian University, Dalian, Liaoning, China; ^3^ Department of Medical Oncology, The Second Affiliated Hospital of Dalian Medical University, Dalian, China; ^4^ Department of Laparoscopic Surgery, The First Affiliated Hospital of Dalian Medical University, Dalian, Liaoning, China

**Keywords:** liver cancer, prognosis, immune cell, cell marker, deep learning

## Abstract

**Introduction:**

Monitoring the response after treatment of liver cancer and timely adjusting the treatment strategy are crucial to improve the survival rate of liver cancer. At present, the clinical monitoring of liver cancer after treatment is mainly based on serum markers and imaging. Morphological evaluation has limitations, such as the inability to measure small tumors and the poor repeatability of measurement, which is not applicable to cancer evaluation after immunotherapy or targeted treatment. The determination of serum markers is greatly affected by the environment and cannot accurately evaluate the prognosis. With the development of single cell sequencing technology, a large number of immune cell-specific genes have been identified. Immune cells and microenvironment play an important role in the process of prognosis. We speculate that the expression changes of immune cell-specific genes can indicate the process of prognosis.

**Method:**

Therefore, this paper first screened out the immune cell-specific genes related to liver cancer, and then built a deep learning model based on the expression of these genes to predict metastasis and the survival time of liver cancer patients. We verified and compared the model on the data set of 372 patients with liver cancer.

**Result:**

The experiments found that our model is significantly superior to other methods, and can accurately identify whether liver cancer patients have metastasis and predict the survival time of liver cancer patients according to the expression of immune cell-specific genes.

**Discussion:**

We found these immune cell-specific genes participant multiple cancer-related pathways. We fully explored the function of these genes, which would support the development of immunotherapy for liver cancer.

## Introduction

Immunotherapy for liver cancer is a new therapy in recent years, which can significantly prolong the survival period of patients with liver cancer and improve the prognosis of patients (1). In particular, combined immunotherapy has benefited more and more patients with liver cancer. For example, the median survival period of the natural course of patients with advanced hepatocellular carcinoma is about 8 months. After the combined treatment of atelizumab and bevacizumab, the life expectancy of patients with advanced hepatocellular carcinoma has more than doubled, and the prognosis of patients has been significantly improved (2). The clinical advantages of immune checkpoint blocking include but are not limited to continuous anti-tumor immune response, relatively weak side effects, low recurrence rate, and even complete remission of some advanced cancers (3). Therefore, immunocheckpoint blocking has been approved for treatment of some cancers. For hepatocellular carcinoma, the blocking of immune checkpoint showed drug resistance in some patients (4). In particular, the combination of anti PD-L1 antibody altazol monoclonal antibody and VEGF neutralizing antibody bevacizumab has become the standard for first-line treatment of liver cancer (5, 6).

However, the proportion of hepatocellular carcinoma patients benefiting from immunotherapy is still very limited. There are many reactions that inhibit the anti-tumor effect of the body at the same time in the tumor microenvironment, which play a certain role in inhibiting the immune cells that play an anti-tumor activity. For example, CD8+T cells often show a dysfunctional state and change into depleted T cells, which cannot effectively eliminate tumor cells and promote the occurrence of immune escape reaction of tumors; In addition, depleted T cells also express multiple inhibitory receptors such as programmed death protein 1 (PD1) and cytotoxic T-lymphocyte associated protein 4 (CTLA4), which drive the progression of liver cancer (7). While other immunotherapies, such as adoptive T cell transfer, vaccination or viral therapy, have not yet shown consistent clinical activity, it is challenging to establish gold standard biomarkers for immunotherapy benefits (8). Therefore, it is still necessary to more systematically and comprehensively study the molecular mechanism of liver cancer immune escape, deeply study the tumor immune response and the dynamic evolution process of microenvironment before and after treatment, and find more specific immune targets and treatment prediction markers to achieve personalized and precise treatment of patients and further improve the therapeutic effect of liver cancer.

At present, some studies have described the key role of inflammation-related genes (IRGs) in regulating the systemic immune response. Improving the understanding of IRGs will help to clarify the mechanism of immunotherapy for liver cancer. At the same time, some key IRGs can also be used as biomarkers to predict the prognosis of liver cancer patients. Chew et al. (9) Genome sequencing technology and bioinformatics can jointly analyze different experimental sequencing data, breaking the limitations of experimental research caused by small sample size, technical limitations and other factors that lead to differences in experimental results. Through integrated bioinformatics analysis of gene expression data, clinical information data and other data, genes related to the occurrence and development of cancer can be mined, which is useful for finding the pathogenesis of cancer and new therapeutic targets, Prognostic biomarkers are of great clinical value. Hu et al. (10) used Robust Rank Aggregation (RRA), Weighted Gene Co expression Network Analysis (WGCNA) and multivariate Cox regression analysis to determine three biomarkers, YWHAB, PPAT and NOL10, which have high diagnostic and prognostic value for liver cancer. Li et al. (11) used immunohistochemistry (IHC) staining and Western blot analysis to find that the expression level of NCSTN in 108 liver cancer tissues was higher than that in adjacent normal tissues. The mRNA information of 364 liver cancer tissues and 50 tissues obtained from TCGA database was consistent; Further research shows that NCSTN passes through β- The activation of catenin promotes the growth and metastasis of liver cancer cells, suggesting that NCSTN may be a potential prognostic marker of liver cancer. Another study used TCGA RNA sequencing data to screen DEGs through bioinformatics analysis, and DEGs were used for GO function enrichment analysis and KEGG pathway enrichment analysis. It was found that a large number of DEGs were involved in cell cycle, molecular processes related to cell division and growth regulation, which were crucial to the occurrence of liver cancer (12).

However, these efforts tend to identify more inflammation-related genes related to the prognosis of liver cancer or predict the occurrence and development of liver cancer through genes. Few articles have reported the relationship between immune related genes and metastasis of liver cancer. Although cancer metastasis can be detected by means of imaging, the exact imaging evidence appears in the late stage of cancer. Predicting cancer metastasis is particularly important for the survival of patients. Some researchers try to predict cancer metastasis by serum biomarkers, but biomarkers such as protein or metabolism interact with each other and are easily affected by environmental factors (13), and the prediction results are unstable. Compared with these biomarkers, gene expression is relatively stable, so it is a feasible idea to predict cancer metastasis by gene (14). The difficulty of this idea lies in finding suitable genes and modeling prediction. With the maturity of single cell sequencing technology, more and more genes with differential expression in immune cells have been identified (15). We call these genes immune cell specific genes. Because the development of liver cancer is closely related to the immune process, immune cell-specific genes have the potential to become a tool for predicting cancer metastasis.

Survival time is another key concern of liver cancer patients. Cox regression is the most common used method in this field. Klempnauer (16) et al. calculated the actuarial survival rate of patients using the product limit method (Kaplan Meier, KM) to explore the prognostic factors after ampullary carcinoma resection, and used Cox proportional risk model to evaluate the correlation between continuous variables and patient survival rate, as well as the simultaneous correlation between multivariate and survival rate. The results showed that the tumor size and grading were statistically significant in terms of patient survival. Datema et al. (17) used the head and neck cancer data to compare the survival rate using Cox regression model and random survival forests (RSF). The results showed that the error rates of RSF and Cox models were similar, but Cox models performed slightly better. Wang et al. (18) obtained gene expression data of LIHC from TCGA, conducted differential expression gene (DEG) analysis and univariate Cox regression analysis to determine DEG related to overall survival rate, and established immune prognosis model by LASSO and multivariate Cox regression analysis. The survival model was evaluated by the size of AUC. In this literature, the AUC of 1-year and 3-year overall survival were 0.781 and 0.783, respectively. Gao et al. (19) integrated the DEG of samples from the LIHC dataset of TCGA and immune related genes from the database InnateDB and ImmPort, and identified immune related differentially expressed genes. Subsequently, candidate prognostic genes were identified by weighted gene co expression network analysis (WGCNA). Cox, LASSO and proportional risk models were used to identify immune related prognosis models, and AUC was used to evaluate the overall survival time prognosis model of survival models. AUC of ROC curve is 0.608 in one year, 0.614 in three years and 0.620 in five years. In the prognostic classification model, Somaya Hashem et al. (20) used the data from the Egyptian National Viral Hepatitis Control Committee and the multidisciplinary liver cancer clinic of Cairo University’s Casel Aini Hospital to establish a prediction model of chronic hepatitis C (CHC) using machine learning technology. The data set of 4423 patients with CHC was investigated to determine the important parameters that predict the existence of CHC. The classification and regression tree, alternating SVM, reducing pruning error tree and linear regression algorithm are used to build a liver cancer classification model for predicting the existence of liver cancer. Results: Statistics showed that age, AFP, ALP, albumin and total bilirubin attributes were correlated with CHC. The model evaluation index is AUROC, and the overall accuracy of AUROC is between 93.2% and 95.6%. The data set used by Maniruzzaman et al. (21) was derived from the 2009-2012 diabetes data set of the National Health and Nutrition Survey (NHANES). LR was used to identify the high-risk factors of diabetes, and a ML based diabetes disease prediction system was adopted and proposed. Naive Bayes (NB), decision tree (DT), Adaboost and RF were used to predict diabetes disease. For performance evaluation, accuracy, sensitivity, specificity, positive predictive value, negative predictive value and area under the curve are used for evaluation. The results show that the overall accuracy of the ML based system is 90.62%, and the combination of LR based and RF based classifiers performs better. This combination will be very helpful to detect diabetes.

The survival time is affected by many factors. Cox regression, a traditional method, cannot well meet the complex nonlinear relationship between gene expression and survival time. However, it can still be used as an important reference tool to determine the correlation strength between genes and survival time. In recent years, deep learning has developed rapidly and has replaced many traditional methods and widely used in many fields. Therefore, it is a feasible idea to use deep learning methods to find the association between immune cell-specific genes and the survival time of liver cancer patients.

## Method

### Marker gene selection

Firstly, we need to obtain immune cell marker genes in liver. CellMarker database has contained the information about this part so we can directly download data from this database (22). Then, we also need to ensure all the marker genes are associated with liver cancer. Therefore, we have to filter some irrelevant genes by DisGeNet database which records associations between genes and diseases (23). Finally, we only select genes that are both related to liver cancer and immune cell marker genes.

### Dimension reduction method

Gene expression was used to predict the metastasis and survival time of liver cancer. Due to the large number of genes, dimension reduction method was needed to be introduced. We chose to use principal component analysis (PCA) to implement dimension reduction since it can fully use information of all genes. Compared with LASSO which is another dimension reduction method, PCA can fuse the expression of all genes, and it is a linear dimensionality reduction method with the least loss of original data information. However, LASSO would cut some genes off to achieve dimension reduction. In this paper, the dimension would shrink from 173 to 90 by PCA, which can retain 99.5% of information.

### Liver cancer metastasis prediction method

Deep Neural Network (DNN) was implemented to predict metastasis of liver cancer. DNN is a multi-layer unsupervised neural network, and uses the output features of the previous layer as the input of the next layer for feature learning. After layer by layer feature mapping, the features of the existing space samples are mapped to another feature space, so as to learn to have a better feature representation of the existing input. The depth neural network has multiple nonlinear mapping feature transformations, which can fit highly complex functions.

The local model of DNN is:


(1) O=σ(∑wx+b)

Where *O* is output and *σ*() is activation function. *w* represents weight and *b* represents bias.

The structure of our DNN model is shown as **Table 1**.

**Table 1:** The structure and parameters of DNN model.

Layer	Details
0	Fully connected layer (units =64)
1	Batch normalization layer
2	Activation layer (ReLu)
3	Fully connected layer (units =64)
4	Batch normalization layer
5	Activation layer (ReLu)
6	Fully connected layer (units =16)
7	Batch normalization layer
8	Activation layer (ReLu)
9	Fully connected layer (units =8)
10	Batch normalization layer
11	Activation layer (ReLu)
12	Fully connected layer (units =2)

### Survival time prediction method

Cox regression was used to do survival analysis. The correlation between genes and survival time can be explored by this method. Cox regression can analyze samples containing missing data, and does not require the survival time distribution type of samples. Cox model, which takes survival outcome and survival time as dependent variables, can simultaneously analyze the impact of multiple factors on survival outcome, and is one of the most widely used survival analysis methods.

Cox proportional risk model assumes that covariates and risks are multiplicative, and the calculation formula of its risk function is as follows:


(2) $$h(t|x_{i})=h_{0}(t)\exp(x_{i}\beta )$$

x=(*x*_*i*1_,*x*_*i*2_,….,*x*_*in*_) represents covariates that may be related to survival time. The factors may be qualitative or quantitative, and do not change with time. *h*_0_(*t*) represents the baseline risk function, that is, the basic risk rate when all risk factors are 0. The baseline risk is unknown, but it is assumed that it is linearly related to *h*(*t*|*x*_*i*_) . *β*=(*β*_*i*1_,*β*_*i*2_,….,*β*_*in*_) represents the partial regression coefficient of the Cox model, which is a group of unknown parameters and needs to be estimated according to the actual sample data.

Since we need to explore whether the expression of the gene is associated with survival time, we cannot use PCA to reduce the dimension of gene expression. The process of DNN prediction of liver cancer metastasis is shown as **Table 2**. The expression of all genes are input into cox regression to determine the hazard ratio and P value of it. In addition, we also used DNN model to predict survival time of liver cancer patients based on the expression of immune cell marker genes. Compared with cox regression, DNN has more powerful nonlinear data processing ability, and can accurately find out the relationship between the expression of multiple genes and survival time, so as to establish a model to accurately predict the survival time of patients through gene expression.

**Table 2:** Process of DNN prediction of liver cancer metastasis.

Input: the number of layers: *L*, Node of each layer activation function of each layer, iteration step *a* , The maximum number of iterations *n* and iteration threshold to stop *ε*, liver cancer samples for training.
Output: *w* and *b*. 1 Initialize *w* and *b* 2 for *i* = 1 to *n* 3 for *j* = 1 to *m* 4 *a* ^1^=*x*_^*i*^_ 5 for *l* = 2 to *L* 6 *a* ^*i*,*l*^=*σ*(*w* ^*l*^ *a* ^*i*,*l*−1^+*b* ^*l*^) 7 Computing the output layer by the loss function *δ* ^*i*,*L*^ 8 for *l* = *L* to 2 9 backpropagation calculation *δ* ^*i*,*l*^=*w* ^*l*+1^ *δ* ^*i*,*l*+1^⊙*σ*(*w* ^*l*^ *a* ^*i*,*l*−1^+*b* ^*l*^) 10 for *l* = 2 to *L*, update *w* ^*l*^ and *b* ^*l*^ : $w^{l}=w^{l}-a\sum_{i=1}^{m}\delta^{i,l}{\left(a^{i,l-1}\right)}^{T}$
$b^{l}=b^{l}-a\sum_{i=1}^{m}\delta^{i,l}$
11 If both a and b changes are less than the threshold *ε*, stop. 12 Output *w* and *b*

**Figure 1:**
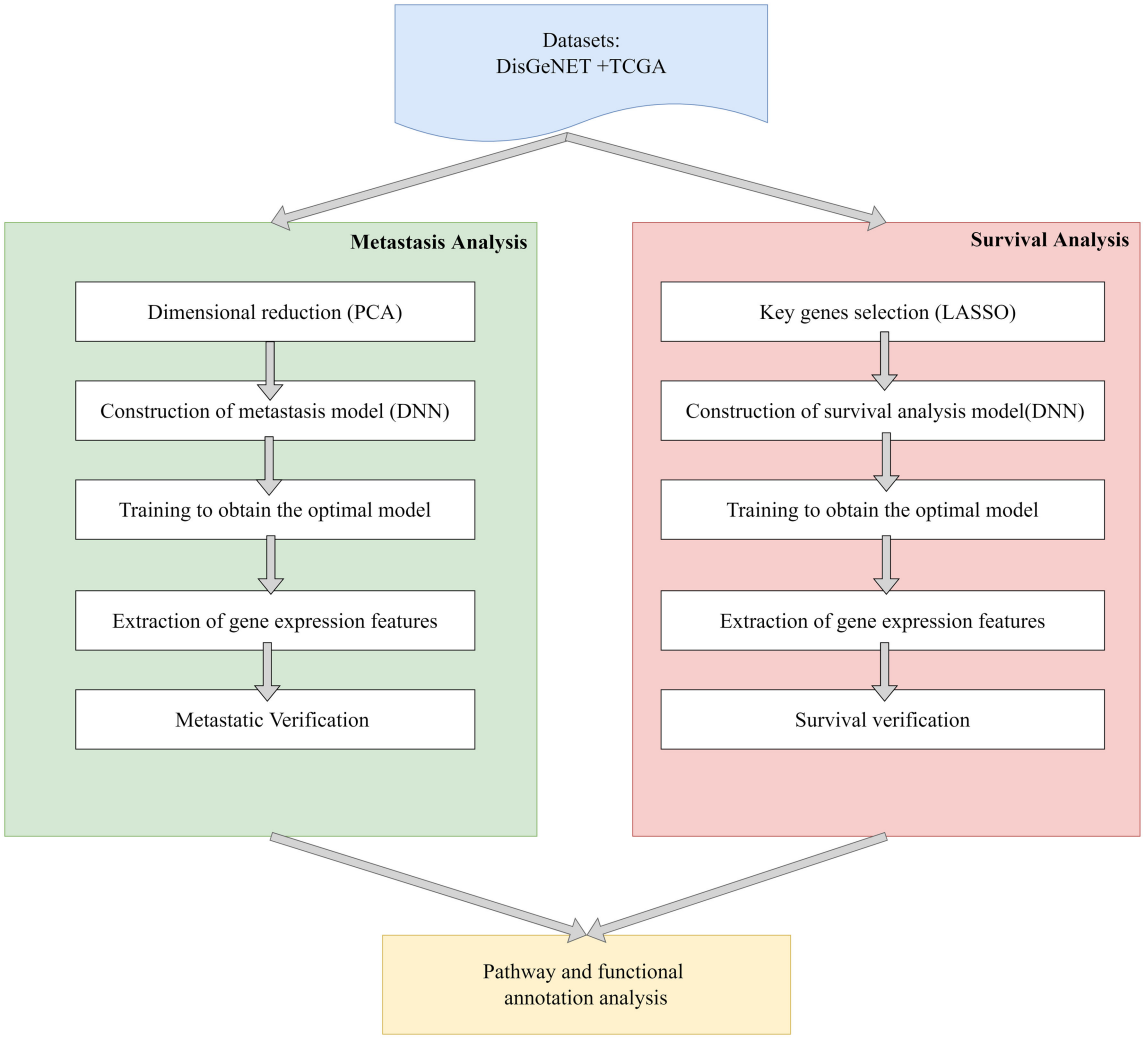
Workflow of prediction.

To predict liver cancer metastasis and survival time, we fused PCA/LASSO with DNN. The whole process of our method is shown as **Figure 1**.

## Result

### Liver cancer-related Immune cell marker genes

**Figure 2:**
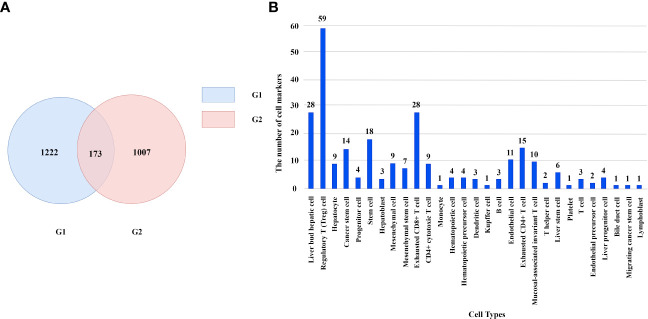
**(A)** The number of genes in different gene sets. **(B)** The number of marker genes corresponding to immune cells.

We obtained 1222 liver cancer-related genes from DisGeNet and we call this gene set G1. In addition, we downloaded cell marker genes from CellMarker database and selected liver tissue to get marker genes in liver cells. By filtering out genes unrelated to immunity, we obtained 1007 immune cell marker genes and we call this gene set G2. There are 173 genes in the intersection of G1 and G2 as shown in **Figure 2A**. These genes correspond to 29 types of immune cells. Regulatory T cell has the most marker genes, reaching 59. Other cell types such as lymphoblast, Platelet and bile duct cell etc. have only one marker gene. The number of marker genes corresponding to immune cells is shown as **Figure 2B**.

These genes will be used to predict the metastasis of liver cancer and the survival time of patients with liver cancer.

### Immune cell marker genes showed strong power in prediction of liver cancer metastasis

**Figure 3:**
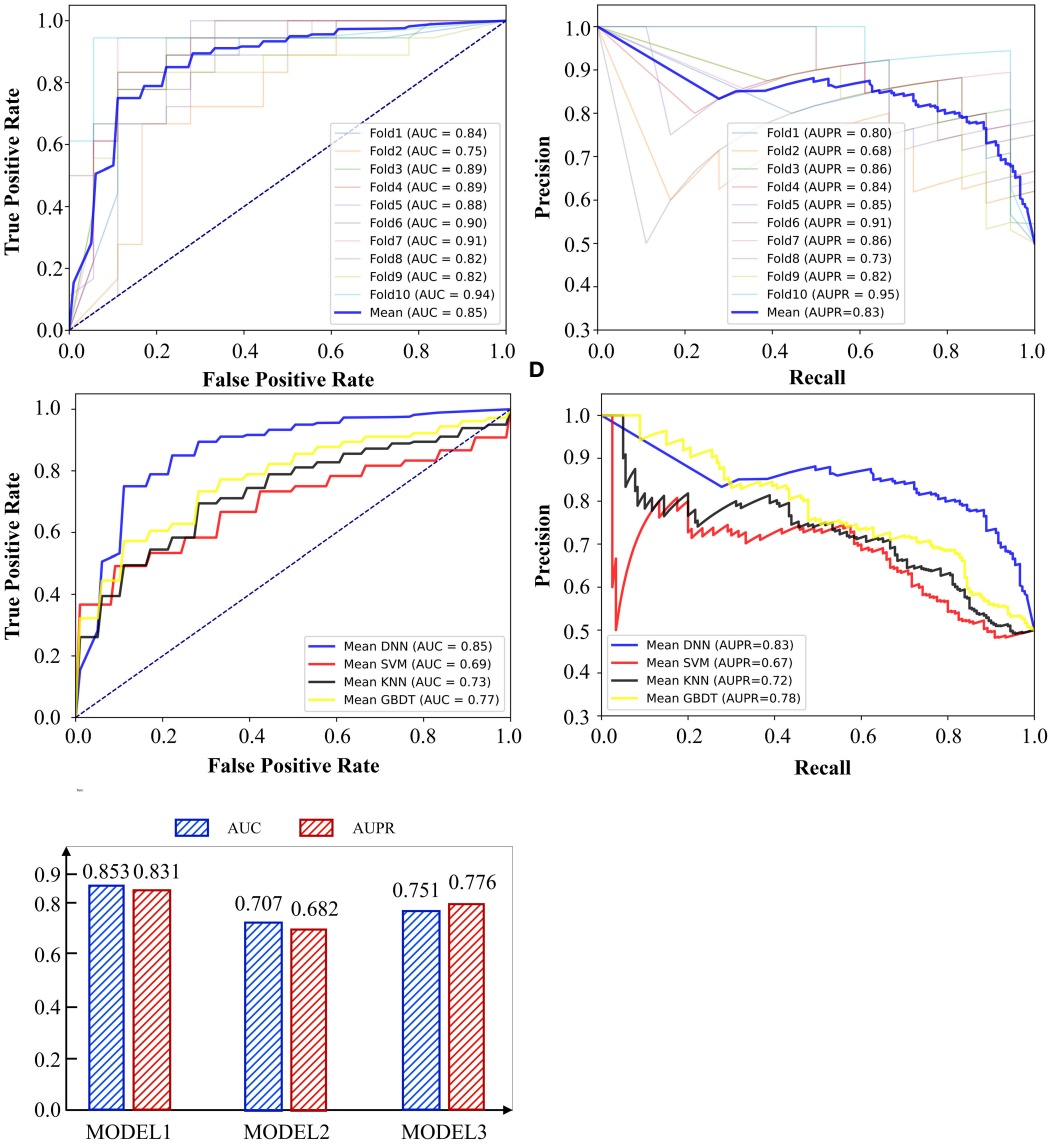
**(A)** AUC curve of 10-cross validation by DNN. **(B)** AUPR curve of 10-cross validation by DNN. **(C)** AUC and AUPR of using different gene sets by DNN. **(D)** AUC curve of different methods. **(E)** AUPR curve of different methods.

372 liver cancer patients with their gene expression have been obtained from TCGA. Among them, 173 patients occurred metastasis. Firstly, we used DNN to predict whether a liver cancer patient would metastasis. 10-cross validation was implemented to verify the accuracy of DNN. To complete this test, we classified all patients into 10 groups. Nine of 10 groups samples were used to train the DNN model and the rest one was used to test the model. After repeating this process by 10 times, each group has been tested once and trained by 9 times. As shown in **Figures 3A, B**, the AUC and AUPR of 10 times tests showed fluctuations, which is caused by the small sample set. The highest AUC reached 0.94 and the lowest one reached 0.75. The average of AUC is 0.85 and AUPR is 0.83, which means it is effective to use DNN to predict the metastasis of liver cancer through 173 immune cell marker genes.

We call the model of predicting the metastasis of liver cancer through 173 immune genes as model 1. In the previous section, we also constructed G1 and G2. G1 contains 1222 genes which are all related to liver cancer. G2 contains 1007 genes which are all immune cell marker genes. We used G1 to train the DNN model and constructed model 2 and constructed model 3 by G2. As shown in **Figure 3C**, model 1 performed best in both AUC and AUPR among all models and model 2 performed worst. This indicates that many genes unrelated to cancer metastasis are mixed in the genes related to liver cancer (G1). These unrelated genes may be related to pathogenesis, but they cannot be well applied to predict cancer metastasis. In addition, immune-related genes have shown a strong ability to predict cancer metastasis, although some of them are even unrelated to liver cancer. G3 has absorbed the essence of two gene sets and achieved the highest accuracy.

We also compared DNN with other methods to show the superiority of it. We compared it with Support Vector Machine(SVM), K Nearest Neighbor(KNN) and Gradient Boosted Decision Tree (GBDT). SVM was selected since it has advantages in small samples. GBDT was chosen since it is a method of reinforcement learning, which is different from SVM and DNN. GBDT, SVM and DNN are all methods based on supervised learning, but KNN is an unsupervised learning method. Therefore, we selected three methods with their own advantages and characteristics to compare with DNN. The results were shown in **Figures 3D, E**. DNN performed best among all methods and SVM performed worst. GBDT is slightly better than KNN. Although SVM is suitable for small sample modeling, the feature dimension of this experiment is high. SVM needs to map features into high-dimensional space and then do regression, so it is difficult to guarantee the accuracy of SVM under high-dimensional features. Although the sample size of this experiment is not high, deep learning method still stands out among other methods with flexible and scalable network layers and nodes.

### Survival time prediction

**Figure 4:**
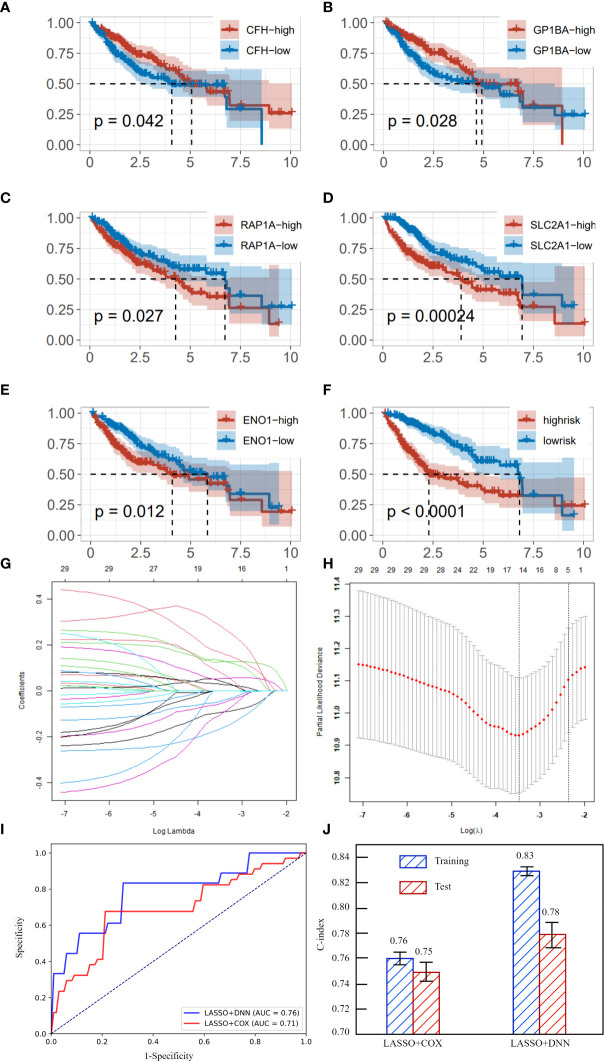
**(A)** survival curve in different expression of CFH. **(B)** survival curve in different expression of GP1BA. **(C)** survival curve in different expression of RAP1A. **(D)** survival curve in different expression of SLC2A1. **(E)** survival curve in different expression of ENO1. **(F)** survival curves of high risk and low risk. **(G)** coefficients of gene under different lambda. **(H)** partial likelihood deviance under different lambda. **(I)** The AUC curves of LASSO+DNN and LASSO+COX regression. **(J)** C-index and its standard deviation of different methods.

Immune cell marker genes were also used to predict the survival time of liver cancer patients. After selecting lambda of LASSO, we obtained five genes that are significantly related to the survival time of liver cancer. They are CFH, GP1BA, RAP1A, SLC2A1 and ENO1. Cox regression was used to test whether the different expression of these five genes would cause significant differences in the survival curve. We divided the training set samples into high expression group and low expression group according to the median of the expression of five genes, and carried out KM survival analysis. As shown in **Figures 4A–E**, the survival time under high gene expression is significantly different from that under low gene expression. The P value are 0.042, 0.028, 0.027, 0.00024, 0.012 respectively, which are all lower than 0.05. It is noteworthy that the high expression of CFH and GP1BA plays a protective role, while the high expression of other genes will reduce the survival time. The regression coefficients of the five best prognostic genes were obtained through the multiple Cox proportional risk regression model, and the expression levels and coefficients of each gene were combined by linear combination to obtain the risk scoring formula: Risk score= -0.19231*CFH+0.5966*RAP1A+0.22919*ENO1-0.19329*GP1BA+0.12790*SLC2A1. **Figure 4F** shows the survival curves in high risk and low risk. The P value of the two survival curves is lower than 0.0001, which means that these five genes can significantly distinguish the survival time of patients. **Figures 4G, H** showed the process of training LASSO to find suitable Lambda.

After obtaining key immune cell marker genes to predict survival time, DNN was implemented to achieve a high precision. We speculate that there is a complex nonlinear relationship between these five genes and survival time, so COX regression cannot achieve the highest accuracy in predicting survival time. We use two error indicators AUC and C-index to evaluate the two models. As shown in **Figures 4I, J**, the AUC of DNN is 0.05 more than that of COX. The C-index in training and testing of DNN are all significant higher than COX. Therefore, we can predict the survival time of patients with liver cancer relatively accurately by the expression of immune cell-specific genes.

### Function analysis of immune cell marker genes

Since we used 173 genes to predict the metastasis and survival time of liver cancer patients and the experiments results showed high precision, these 173 genes should be significantly associated with the prognosis of liver cancer in function. Therefore, we explored the pathway and GO terms of these genes.

Enrichment analysis showed that these genes were significantly associated with 83 pathways and 1430 GO terms (P <0.05). These pathways can be divided into five classes: 2 Metabolism, 11 environmental information processing, 5 Cellular Processes, 10 Organismal Systems, 55 Human Diseases. The GO terms can be divided into three classes: 1309 biological process, 41 cellular component, 80 molecular function.

**Figure 5:**
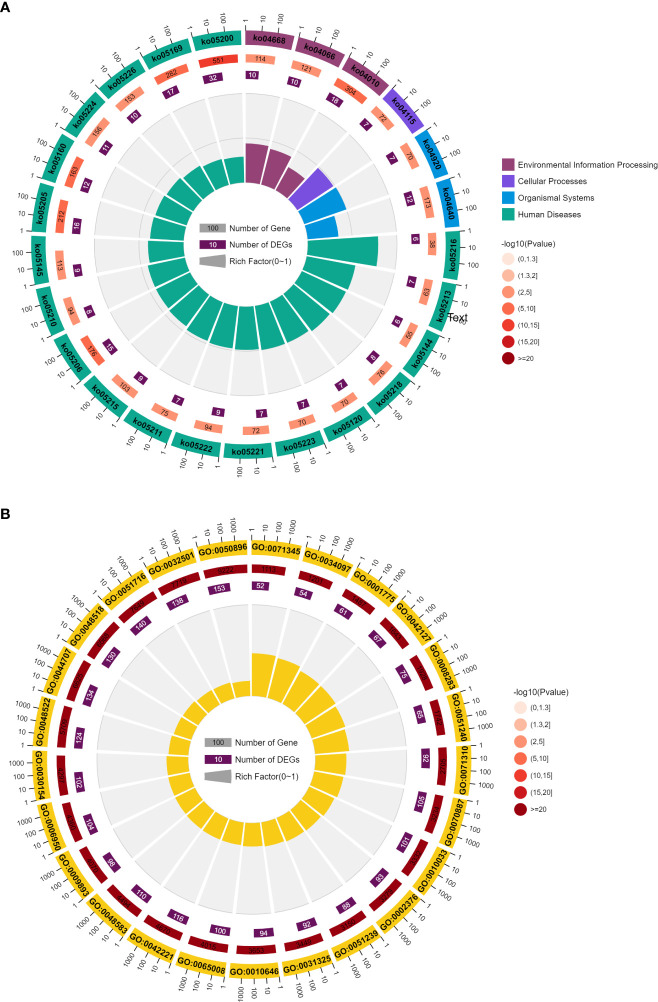
**(A)** Top 25 KEGG of immune cell marker genes. **(B)** Top 25 GO analysis of immune cell marker genes.

**Figure 5** shows the top 25 pathways and GO terms of immune cell marker genes. For the KEGG pathways, these genes enriched processes associated with 3 environmental information processing, 1 cellular processes, 2 organismal systems and 19 human diseases pathways. Among these pathways, the most significant pathway is ko05200. 32 of 173 immune cell marker genes enriched in this pathway which is a cancer-related pathway. In addition, immune cell marker genes also significant enriched in k005160 pathway which is Epstein-Barr virus (EBV) infection. EBV has been proved to be associated with a wide range of human malignant tumors, and it can induce abnormal gene expression during latent infection. The expression of these genes can effectively identify three different latent patterns related to cancer types. Li et al. (24) reported that EBV infection exists in liver cancer tissue, and EBV may be involved in the occurrence of liver cancer. At the same time, HDV does not appear to play a direct role in hepatocellular carcinogenesis(HCC). Kang et al. (25) found that HCCs with immune cell stroma (HCC-IS) are a distinct HCC subtype associated with a favorable prognosis and frequent EBV-positive tumor-infiltrating lymphocytes (TILs). Paradoxically, however, a high density of EBV-positive TILs in tumors was associated with poorer prognostic outcomes. HCC-IS patients may be candidates for immunotherapy.

Another important pathway is ko04010 which is a MAPK signaling pathway. This pathway is closely related to cell proliferation, differentiation and migration. This path is in the cluster of signal transduction related pathways. There are direct connections with 15 of the 35 core channels. In the entire KEGG database, there are as many as 104 types of pathways directly related to MAPK pathway, ranking first among the pathways with subdivided functions. MAPK pathway is almost one of the newest core pathways in the intracellular regulatory network. If 104 pathways are classified according to class B, the largest proportion is 21 cancer-related pathways and 15 infectious disease related pathways. It can be seen that MAPK pathway is mainly related to cancer and immune/inflammatory diseases. Multiple studies have reported the high relationship between MAPK pathway and prognosis of liver cancer. Although the mutation frequency of the MAPK/ERK signaling pathway is low, frequent activation of the signaling pathway was found in HCC patients (26). MAPK/ERK signal transduction has prognostic significance, and it has been reported that the increased expression level of RAS effectors is highly correlated with the low survival rate of HCC patients (27). Meanwhile, RAF-1 overexpression is also considered as an independent marker of early tumor recurrence and poor prognosis (28). Based on MEK/ERK expression and phosphorylation, MAPK/ERK signaling is thought to be activated in approximately 50% of early-stage HCC patients and almost all advanced-stage HCC patients (29). Most of the 173 genes were related to GO:0050896, reaching 153 genes. The Synonyms of this term is physiological response to stimulus. Multiple studies have reported the association between GO:0050896 and liver cancer (30, 31). Romayor et al. (32) found silencing of sinusoidal DDR1 would affect the genes in GO:0050896 and is strongly related to the liver metastasis of colon carcinoma.

CFH is the marker of Natural killer T (NKT) cell and Liver bud hepatic cell. RAP1A is the marker of Regulatory T (Treg) cell. ENO1 is the marker of Megakaryocyte cell. GP1BA is the marker of Regulatory T (Treg) cell. SLC2A1 is the marker of Cytotoxic CD4+ T cell. Kalathil et al. (33) reported that the frequency of circulating and tumor-infiltrating NK cells is positively associated with survival benefit in hepatocellular carcinoma (HCC) and has prognostic significance, suggesting that functional impairment of NK cells is closely related to HCC progression. Wang et al. (34) analyzed the frequency of Tregs in liver cancer by flow cytometry and immunohistochemistry. Experimental results show that HCC patients have a high frequency of Tregs, and a high number of Tregs is associated with poor prognosis. Hepatocellular carcinoma cells induce Treg production by secreting TGF-β1. *In vivo* experiments showed that knockdown of TGF-β1 reduced the number of Tregs and metastatic nodules in mice. Although the role of megakaryocytes in the metastasis of liver cancer has not been reported, its role in the metastasis of other cancers has been reported. Raphael Leblanc and Olivier Peyruchaud (35) show that increasing the number of megakaryocytes (MK) in the bone marrow results in a high bone mass phenotype and inhibits skeletal metastases formation in prostate cancer cells. Zhao et al. (36) found that CD4+plays a crucial role in the survival of HCC through a retrospective cohort study of 170 BCLC-B HCC patients (42 HIV+).

## Discussion

There are many reactions in the tumor microenvironment that inhibit the anti-tumor effect of the body and play a certain role in inhibiting the immune cells that play the anti-tumor activity. For example, CD8+T cells often display a state of dysfunction and turn into depleted T cells, which cannot effectively eliminate the tumor cells and promote the immune escape reaction of the tumor; In addition, depleted T cells also express programmed death protein 1 (PD1) and cytotoxic T-lymphocyte-associated protein 4 (CTLA4) and other inhibitory receptors, driving the progression of liver cancer (37). Therefore, it is still necessary to study the molecular mechanism of immune escape of liver cancer more systematically and comprehensively, and to further study the dynamic evolution of tumor immune response and microenvironment before and after treatment. Because the progress of liver cancer is closely related to immunity, this paper screened immune cell-specific genes, and associated the expression of these key genes with the prognosis of liver cancer through in-depth learning method, so as to accurately predict the metastasis and survival time of liver cancer.

In the experiment of predicting liver cancer metastasis by selected 173 immune cell specific genes, the DNN model obtained the results of AUC=0.853 and AUPR=0.831, which are significantly superior to other algorithms. At the same time, we also established two models for predicting the metastasis of hepatocellular carcinoma by all immune cell-specific genes and liver cancer-related genes respectively. The experimental results show that the accuracy of these two models is lower than that of the models established by selected 173 immune cell specific genes. This indicates that not all genes that are differently expressed in immune cells are related to the prognosis of liver cancer, and not all genes related to liver cancer are related to the prognosis of liver cancer. Only by accurately identifying and classifying genes related to pathogenesis, prognosis and treatment can achieve personalized and precise treatment.

Selected 173 immune cell specific genes were also used to predict the survival time of liver cancer patients. Cox regression and LASSO were implemented to explore the significant genes related to survival time. Among the 173 genes, the P value of five genes are lower than 0.05. We discussed the difference in survival curves for high/low expression of these three genes. Previous studies also reported the strong association between these genes and prognosis of liver cancer. Solute carrier family 2 member 1 (SLC2A1) is a key factor for glucose transport and metabolism in cancer cells (38). Yang et al. (39) used a collection of 50 primary liver cancers and 100 liver metastases to explore tumor immune microenvironment and found that SLC2A1 showed a negative correlation with T cell infiltration. Additionally, Fang et al. (40) found that overexpression of SLC2A1 promotes cell survival, migration, and invasion to affect the progression of hepatocellular carcinoma. These studies show that SLC2A1 exhibits a vital role in the occurrence and development of tumor and may serve as a promising marker for the prognosis and treatment of liver cancer; Alpha-enolase is proved to be related to tumor cell division, proliferation, apoptosis and metastasis (41, 42). Jiang et al. (43) demonstrated that ENO1 is upregulated in liver cancer cells or tissues and it is a key factor of promoting growth and metastasis of liver cancer. Besides, Zhang et al. (44) found that ENO1 influences the survival of liver cancer cell by suppressing iron regulatory protein 1 (IRP1) and revealed the importance of ENO1-IRP1-Mfrn1 pathway in the pathogenesis of liver cancer; Glycoprotein Ib platelet alpha subunit (GP1BA) is a glycoprotein involved in platelet adhesion. Sun et al. (45) found that genes implicated in GP1BA and other formation of tumor cell-platelet microaggregates showed increased expression from hepatic vein to peripheral artery and retains their elevated expression level in peripheral artery and peripheral vein through studies on CTCs of HCC patients; Complement factor H (CFH) is a critical regulatory protein of the complement alternative pathway (AP). Seol et al. (46) analyzed primary liver tumorsphere and found CFH and other complement protein was significantly up-regulated and the knockout of CFH expression eliminated the formation and induced differentiation of tumor cells, while the over expression stimulated the expression of stem cell factors and the growth of cells *in vivo*. Laskowski et al. (47) found that the increase of CFH mRNA expression is related to the improvement of survival rate of HCC patients, while the CFH mutation is related to the poor survival rate. These studies show that CFH is essential to control the activation of complement in the liver and may represent a new diagnostic and prognostic marker of liver cancer; RAS-associated protein 1 (RAP1) is a member of telomere-binding proteins, which has been proved to be related to the occurrence, development and chemoresistance of human cancer (48). Ferrara-Romeo et al. (49) found that Rap1 -/- females are more susceptible to DEN-induced liver injury and hepatocellular carcinoma (HCC) and RAP1 deficiency will lead to the increase of liver cancer after the occurrence of chemical liver cancer. In addition, Zha et al. (50) demonstrated that knocking down Rap1 significantly enhanced the apoptosis of HepG2 cell line and the chemosensitivity of 5-fluorouracil (5-FU) and got the conclusion that targeted RAP1 signal pathway has therapeutic value for liver cancer.

Overall, we screened immune cell-specific genes related to the prognosis of liver cancer, and effectively predicted the metastasis and survival time of liver cancer through these genes. Pathways and gene functions related to the prognosis of liver cancer were discovered, which provides help to reveal the pathogenesis of liver cancer and find more specific immune targets and treatment prediction markers.

## Data availability statement

The datasets presented in this study can be found in online repositories. The names of the repository/repositories and accession number(s) can be found in the article/supplementary material.

## Author contributions

JL, JQ, LX and CQ designed the experiments, analyzed the data, and wrote the manuscript. GS and XL analyzed the bioinformatic data. HH, LX provided important ideas. This whole work is guided by JZ. All authors contributed to the article and approved the submitted version.
